# Novel Development and Prospects in Pathogenesis, Diagnosis, and Therapy of Alzheimer’s Disease

**DOI:** 10.3233/ADR-230130

**Published:** 2024-02-20

**Authors:** Zenghui Teng

**Affiliations:** Medical Faculty, Institute of Neuro- and Sensory Physiology, Heinrich-Heine-University Düsseldorf, Germany

**Keywords:** Alzheimer’s disease, amyloid-β, biomarker, neuroinflammation, tau

## Abstract

Alzheimer’s disease (AD) is the most prevalent neurodegenerative disease with cognitive decline and behavioral dysfunction. AD will become a global public health concern due to its increasing prevalence brought on by the severity of global aging. It is critical to understand the pathogenic mechanisms of AD and investigate or pursue a viable therapy strategy in clinic. Amyloid-β (Aβ) accumulation and abnormally hyperphosphorylated tau protein are the main regulating variables in the pathological phase of AD. And neuroinflammation brought on by activated microglia was found to be one risk factor contributing to changes in Aβ and tau pathology. It is important to investigate the unique biomarkers of early diagnosis and advanced stage, which may help to elucidate the specific pathological process of AD and provide potential novel therapeutic targets or preventative measures.

## INTRODUCTION

As the primary cause of dementia, Alzheimer’s disease (AD) is the most prevalent neurodegenerative disease for which there is no effective treatment available. Short memory loss is the early clinical symptom of AD. Other features of AD include the cognitive decline and behavioral dysfunction at a moderate level [1]. The hallmark clinical aspect of AD is the progressive impairment of behavioral and cognitive abilities, including memory, mood, emotions, language, attention, and judgement. Nearly 60%–80%of AD patients will eventually lead to dementia [2].

Aging is a risk factor for AD. According to the latest findings in epidemiological research, AD will become a global public health concern due to its increasing prevalence brought on by the severity of global aging. Every five years, the number of new instances of AD in adults over 60 doubles. For those over 60, the prevalence rate is 1%, and for those over 85, it is 40%[3]. In the future, more elderly people will be diagnosed with AD due to the increasing illness risk brought on by the aging population.

In 2020, there were more than 50 million people globally who have AD, up from 35 million people who received a diagnosis in 2010. According to the recent Alzheimer’s Disease International report from 2021 [4], 75%of dementia patients worldwide, mostly in low- and middle-income countries, remain undiagnosed due to the limitations of early diagnosis. It is anticipated by 2030, 80 million people will be diagnosed with AD, and by 2050, the number will rise to around 150 million. Furthermore, current studies indicate that one third of men and two thirds of women are at risk of developing AD in their lives. It is yet unknown why women have a higher risk of disease than men, as well as the underlying biochemical mechanisms and motivations. AD is currently estimated to cost $1 trillion globally in medical expenses, which will have a significant negative impact on society’s ability to sustain its economy. Therefore, it is critical to understand the pathogenic mechanisms of AD and investigate or pursue a viable therapy strategy in clinic.

## PATHOGENESIS

According to differing clinical processes and pathogenic variables, AD is separated into early-onset family AD (EOAD) and late-onset sporadic AD (LOAD). Less than 5%of cases of AD are familial (fAD), while sporadic AD accounts for 95%of cases (sAD). Most fAD patients are diagnosed before the age of 65 and have a background of mutations in numerous genes, including *A* β*PP* (amyloid-β precursor protein), *PSEN1* (presenilin 1), and *PSEN2* [5]. EOAD typically includes a family history of inherited disease. LOAD is primarily sporadic and is associated with the multi-risk factors including type 2 diabetes, traumatic brain injury, stroke and *APOE* 4 (apolipoprotein 4) gene. The genetic factor of sAD is *APOE* 4 [6], and sAD is also influenced by sortilin-related receptor 1, clusterin, complement component receptor 1, CD2AP, EPHA1, and MS4A4/MS4A6E. Additionally, there are other factors that can affect the LOAD, including as depression, lower education, and even dietary changes or environmental alterations [7, 8]. It has been also demonstrated that ApoE 4 is produced by astrocytes, and that ApoE 4 can exacerbate the inflammatory response by further stimulating the production of inflammatory chemokines and cytokines. Microglia activation with lipopolysaccharide (LPS) triggers an inflammatory response in a mouse mode that is associated with *APOE* 4 and may worsen neurodegeneration [9].

### Amyloid-cascade hypothesis

**Fig. 1:**
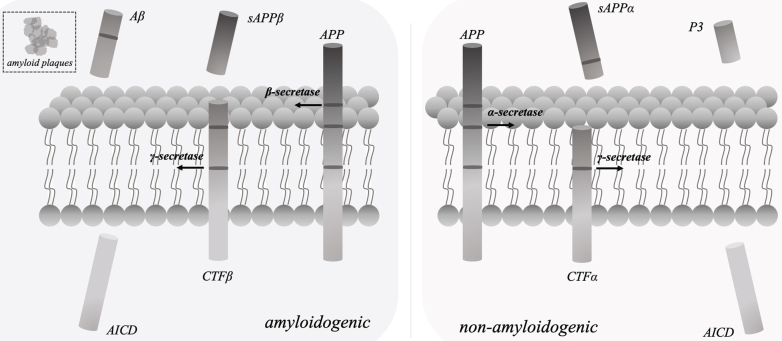
In line with the amyloid-cascade theory of Aβ production, sAβPPβ, Aβ peptides, and the intracellular domain of AβPP (AICD) were released from AβPP by β-secretase and γ-secretase cleavage, respectively. The essential components of γ-secretase include nicastrin, PSEN 1, PEN 2 and APH 1. In the non-amyloidogenic pathway, sAβPPα, P3 peptides, and AICD were produced by α-secretase and γ-secretase cleavage, respectively.

In the pathogenic phase of AD, amyloid-β (Aβ) accumulation and abnormally hyperphosphorylated tau protein are the primary regulatory factors. The development of amyloid plaques was caused by extracellular Aβ accumulation [10], and neurofibrillary tangles were brought on by intracellular tau phosphorylation [11]. And the development of neurofibrillary tangles and amyloid plaques induced the further synapse loss and cognitive dysfunction. In line with the amyloid-cascade theory of Aβ production [12], sAβPPβ, Aβ, and the intracellular domain of AβPP (AICD) were released from AβPP by β-secretase and γ-secretase cleavage, respectively (Fig. 1). γ-secretase [13] consisting of nicastrin, PSEN 1, PEN 2 (presenilin enhancer 2) and APH 1 (anterior pharynx defective 1) as the essential components plays the crucial role in above process.

According to the recent finding on clinical drug trials for AD, γ-secretase enzyme may be a promising therapeutic target for controlling Aβ level regulation [14]. Therefore, specific γ-secretase inhibitors, modulators that particularly reduce of Aβ_42_ production, or stabilizers that produce longer Aβ peptides (≥42) [15], in combination with other therapies that target hyperphosphorylated tau or neuroinflammation, may be used to effectively slow down the pathological progression and development of AD from early to later stages. Such Aβ production, which largely includes Aβ_1–40_ and Aβ_1–42_ in above pathological process of amyloidogenic pathway played the major role in the development of the pathological process of AD [16]. Aβ_42/40_ is currently a critical biomarker of AD clinical diagnosis by positron emission tomography (PET)-computed tomography (CT) or cerebrospinal fluid (CSF) checking [17]. Additionally, mutations in the genes for *A* β*PP*, *PSEN1*, and *PSEN2* could lead to or accelerate the accumulation of Aβ peptides [18], and these alterations were the specific genetic background on fAD etiology.

More studies have recently offered various newly proofs that intracellular Aβ (iAβ) [19], a biomarker or intracellular modulator, may have a great impact on the pathological progression of AD. The production of iAβ, the import of extracellular Aβ, and the clearance of iAβ [20], however, would help to clarify the mechanism of iAβ accumulation and provide more information on the effects of Aβ toxicity on synapse dysfunction. Our latest study [21] also evidenced that γ-secretase inhibitor regulated iAβ toxicity by slowing down endocytosis, which was associated to N-cadherin-CTF, a synaptic cell adhesion molecule. These recent findings may offer novel insight into the molecular mechanism of AD and provide new prospective targets for drug discovery and development. The regulation of synaptic morphology and plasticity was associated with the AβPP [22], which functions as a synaptic adhesion molecule. Other synaptic adhesion molecules, such as cadherin [23], neurexin, neuroligin [24], LRRTM3 [25], and NCAM [26], also have significant effects on AD’s pathophysiology. Additionally, a plausible cause of synapse loss or dysfunction could be an interaction between the synaptic adhesion molecules and Aβ toxicity on the synapses.

These Aβ effects were the main cause of neurodegeneration in familial AD, and Aβ accumulation and aggregation may be responsible for the pathogenic changes in hyperphosphorylated tau protein. Thus, synergistic effect of Aβ and tau should be taken into consideration.

### Synergistic effect of Aβ and tau

Aβ and tau play the crucial roles in the pathological progression of AD and are, in theory, the most significant therapeutic targets for ongoing new medication research in this area. Many options for anti-Aβ deposition and anti-tau protein hyperphosphorylation were able to exhibit good pharmacological effects in pre-clinical investigations [27, 28] in accordance with the needs of drug design by reducing Aβ protein deposition or lowering tau protein hyperphosphorylation, respectively. It would also be in keeping with the original purpose of anti-AD drug creation and might dramatically slow the trajectory of cognitive loss in AD animal models [29]. Although the above-mentioned candidate drugs could still limit the level of tau protein hyperphosphorylation and reduce Aβ deposition in the brains of AD patients. Most clinical trials to far have failed because additional clinical investigations [30, 31] have demonstrated that the decline in cognitive function of these patients has not been significantly reversed or modified.

PET molecular neuroimaging was able to detect both total or hyperphosphorylated tau and Aβ distribution in the brain, it has been proved that the pathological changes of tau protein were independent with Aβ accumulation [32], both tau and Aβ might be occurred in distinct “time windows” of AD development. Additionally, neuroimaging data also demonstrated [33] that such changes to Aβ and tau were seen in several targeted regions during the early stages of AD, with tau showing up earlier among these changes. In the early stages of AD, hyperphosphorylated tau is dispersed alone in the entorhinal cortex of the brain without the presence of Aβ [34]. Such hyperphosphorylated tau detected solely by PET in the particular neurofibrillary tangles in the absence of Aβ accumulation provided the additional proof that the pathological process of tau protein may be the alternative pathway independent of Aβ in the development of AD pathology. Furthermore, it is evident that Aβ accumulation in the late stages of AD could facilitate or enhance tau distribution in the neocortex, promoting AD-related neurodegeneration [35].

As a result, this concept offers the novel insights into the pathological process of sporadic AD, and studying the synergistic interaction between Aβ and tau will help us clarify more details of the mechanism of AD. Furthermore, tau-mediated neurodegeneration, which is independent with the Aβ pathway, has an additional impact on the pathological process of AD [33], which may be part of the reason why anti-Aβ therapies applied solely by only removing Aβ obviously from clinical AD patients were unable to improve or reverse the cognitive decline. In addition, Phosphokinases CDK 5 [36], GSK 3[37], and ERK2 [38], may also control tau protein hyperphosphorylation in sporadic AD.

Even though it has been established that the pathological changes of tau protein were independent of the accumulation of Aβ, the relationship between the two remains controversial. Since the molecular mechanism underlying AD pathogenesis is still unclear, more experimental data and research will be required to investigate it in the years to come. These additional investigations will support a theoretical framework for clarifying the pathological mechanisms and signaling pathways associated with AD, as well as present new pharmacological targets and therapeutic approaches for the clinical treatment of AD patients. Due to the synergistic effects of Aβ and tau discussed above, a combined therapeutic strategy using anti-tau and anti-Aβ may have an anticipated positive impact on the future clinical care of AD patients.

### Neuroinflammation

In addition to the amyloid cascade hypothesis of AD, neuroinflammation brought on by activated microglia was found to be one risk factor contributing to changes in Aβ and tau pathology [39, 40]. Pathological tau protein can induce the production of IL-1 via exacerbating neuroinflammatory reactions. In hippocampus, tau phosphorylation is decreased by inhibiting adenosine A2A receptors [41]. In the rTg4510 model, LPS may lead to the form of neurofibrillary tangles and hyperphosphorylation of tau [42]. Numerous research on the biomarkers for neuroinflammation have shown that the activation of microglia in such an inflammatory response within the brain led to the release or production of cytokines or chemokines such as IL-3, IL-12, or IL-10, etc. In animal models, stimulation of astrocytes and microglia promotes amyloid accumulation and speeds up inflammatory responses through secretion of cytokines. Research has indicated that there is a greater presence of TNF-α and IL-6 in the brains of individuals suffering from clinical AD. Neurotoxic astrocytes may arise in a neuroinflammatory environment due to the activation of microglia and the release of IL-1, tumor necrosis factor (TNF-α). Furthermore, these neurotoxic astrocytes are unable to support the development of new synapses [43, 44].

Recent clinical studies [45] have demonstrated that AD patients who have higher amounts of IL-12 in their brains, even in the presence of a significant quantity of Aβ accumulation concurrently, will not experience severe cognitive impairment. Intriguingly, tau tangles are rarely seen in the brains of AD patients if IL-12 levels are higher. Additionally, raising the levels of IL-12 and IFN-γ may postpone or slow down cognitive loss, but the precise molecular pathways driving the degenerative course of AD are still unknown. The most recent clinical research report also confirmed that IL-3 released from astrocytes in the brain could control the activation of microglia and reprogram neuroinflammation [46], as well as clear the accumulation of extracellular Aβ plaques and reduce the formation of intracellular tau tangles. However, it is still unknown whether this action of IL-3 could regulate or reverse cognitive decline. This means that the pathogenic changes brought on by these cytokines may dramatically restrict the progression of AD. Furthermore, since the development of synapses is the basis of learning and memory, microglia have the ability to alter or remove existing synapses as well as control synaptic plasticity by remodeling the extracellular matrix. The balance between synapse formation and synapse elimination will be regulated by the effect of neuron-glia communication system [47]. Our latest research [48] shown that hemisynapse formation could be induced on cortical or cerebellar astrocytes following interaction with cortical explant axon by overexpression of the synaptic cell adhesion molecules LRRTM2. This finding may offer a potential approach for treating AD by artificially inducing the newly synapse formation. The process of the neuron-glia communication system also involves the secretion or release of IL-33 and alterations in microglia [49]. Age-related alterations in dendritic spines are all linked to IL-33 and its receptor IL1RL1. The loss of dendritic spines that occurs with aging can be stopped by artificially raising IL-33 levels, which encourages synapses’ remodeling and growth. The aforementioned novel biomarker from blood or CSF may be used in the clinical diagnosis or treatment of AD patients.

According to recent breakthroughs in research on AD therapeutic medications, if anti-Aβ therapy is taken as early as feasible in conjunction with the multi-targeted treatments, it may primarily be successful or delay the onset of clinical symptoms. As a result, early diagnosis using a combination of precise techniques like magnetic resonance imaging (MRI), PET, biomarker measurement, or cognitive score evaluation becomes increasingly crucial and urgent in order to prevent the destructive progression of AD from the early stages to the late phases.

## DIAGNOSIS

The diagnosis of AD is based on the cognitive, functional, and behavioral scores of the patient, medical evaluation by doctors, combined with PET or MRI brain imaging, and analysis of biomarkers such as Aβ_42/40_, p-tau, glia fibrillary acid protein, and neurofilament light from CSF (cerebrospinal fluid) or blood.

### Basic brain imaging

The use of MRI [50], which helps to comprehend and observe the changes in brain structure related to AD pathology, is currently one of the key medical examination techniques used in hospital settings for the diagnosis of AD. A prominent clinical hallmark of dementia brought on by AD is the loss of brain tissue, and an MRI will reveal morphological alterations in AD patients, such as the atrophy of specific brain regions. By assessing the distribution of Aβ or phosphorylated tau in different brain regions from preclinical stage or moderate cognitive impairment to dementia of AD, PET imaging [51] can offer the metabolic changes in AD brain. It’s important to elucidate the pathogenic mechanism of neuroinflammation in animal models and AD patients by using PET molecular imaging. The most neuroinflammatory PET imaging targets is mitochondrial 18 kDa translocator protein (TSPO), and the PET tracer ^[18F]^flortaucipir was used to detect tau accumulation and neuroinflammation [52, 53].

### Novel CSF or blood biomarkers

Aβ_42/40_ is one important biomarker for the diagnosis of AD patient through PET-CT or CSF examination [17]. The clinical diagnostic level of AD will be raised by using single molecule array (SIMOA) or mass spectrometry as high sensitivity immunoassays to measure novel biomarkers in patient plasma or CSF. Therefore, creating simple, timely, affordable, and accurate diagnostic methods based on blood samples can aid us in promptly screening AD patients as early as feasible and in taking early preventative action to slow down the clinical pathological process of AD patients. Neurofilament light [54, 55] has been quantified from patient CSF or plasma samples and utilized as a general indicator of neurodegenerative disease to represent neuronal loss or injury in numerous countries to date. Another novel blood-based AD biomarker is glia fibrillary acid protein [56], which was linked to the pathological alterations on Aβ and released during astrocytic activation. New biomarkers from inexpensive blood tests can change the drawback and limitations on CSF investigation caused by the necessity of lumbar puncture, as well as the existing expensive clinical diagnosis of AD patients using PET imaging. It will aid in the development of novel AD therapeutics. Although the patient does not exhibit any signs of cognitive impairment in the preclinical stage of AD, there are nonetheless neuropathological changes in the tau or Aβ proteins. In the laboratory, it has been reported that several new phosphorylated tau specificities, including p-tau 181, p-tau 217, and p-tau 231 [57–59], have been used by research scientists to assess tau and Aβ pathological process as the novel blood biomarkers for the potential clinical diagnostic method of AD.

### Applications of artificial intelligence

Additionally, due to the recent rapid advancement of computational intelligence techniques, computer-aided diagnosis systems for disease using medical artificial intelligence algorithms such as machine learning, deep learning, and convolutional neural network model [60] will provide the most accurate diagnosis and early prevention for AD patients in conjunction with professional physician clinical judgment through the cognitive tests, imaging data, or biomarkers analysis. Using a deep learning algorithm and ^18^F-FDG PET imaging [61], it can predict the AD diagnosis. By applying machine learning in conjunction with drawing characteristics and Montreal Cognitive Assessment scores [62], it has been suggested that early identification and diagnosis of cognitive deficiencies will assist enable preventative dementia intervention in clinic. The Digital Neuro Signature from Altoida was used to assess neurocognitive function on an individual basis [63] and was approved as a breakthrough device by the FDA in August 2021. Therefore, in the next years, a basic clinical early evaluation system may screen cognitive capacities of high-risk groups early on by utilizing deep learning and machine learning algorithms in addition to the Mini-Mental State Examination, drawing clock, AD8 test, and other tests. This will play a certain auxiliary role in the early prevention of AD.

## THERAPIES

AD will unavoidably place significant societal burdens on people’s lives all around the world before the development of efficient treatment medications or procedures. It is very advantageous economically and socially to investigate the pathogenesis of the illness and identify potential new treatment targets or preventative measures (Table 1) in order to lessen clinical symptoms and enhance the quality of life for AD patients [64]. The FDA has approved the use of the therapeutic medications Galantamine, Donepezil, Rivastigmine (AChE inhibitors), and Memantine (NMDA receptor antagonists) to treat the clinical symptoms of AD patients, although none of them reverse or improve cognitive impairment [65]. Additionally, the FDA approved one Aβ immunotherapy using Aducanumab from Biogen in June 2021, while this choice is still causing debate among scientists [66, 67]. In January 2023, Lecanemab was approved by FDA for the treatment of AD through the accelerated approval pathway. It was obtained traditional approval from FDA in July 2023 [68–70] (Table 2).

**Table 1:** Top 5 list for the CADRO target in all phases of clinical trials (*until 1 January 2023*)

CADRO target	Number of agents	Percentage of	Agents	Agents	Agents
	(all clinical phase)	total agents	(phase 1)	(phase 2)	(phase 3)
Transmitter/Receptor	29	15.5%	6	12	11
Inflammation	25	13.4%	6	17	2
Amyloid	25	13.4%	7	11	7
Synapse plasticity	21	11.2%	1	14	6
Tau	14	7.5%	4	8	3

CADRO, Common Alzheimer’s Disease Research Ontology; There were 187 total agents in all phases of clinical trials until 1 January 2023.

**Table 2:** New representative drugs for Alzheimer’s disease

Agent	Name	Company	Mechanism of action	Therapeutic purpose	Status
Aducanumab	Aduhelm	Biogen	Monoclonal antibody	DMT, biologic	Accelerated approval from FDA, *June 2021*
Donanemab	LY3002813	Eli Lilly	Monoclonal antibody	DMT, biologic	Applied for full FDA approval, *June 2023*
Lecanemab	Leqembi	Eisai/Biogen	Monoclonal antibody	DMT, biologic	Traditional approval from FDA, *July 2023*

DMT, disease modifying therapy.

Even though they performed well for AD animal models in the preclinical research stage, the majority of medication candidates undergoing clinical trials are unable to slow down or reverse the clinical symptoms of cognitive loss in individuals with AD. Therefore, novel regenerative therapies using stem cells, such as mesenchymal stem cell or human induced pluripotent stem cell (hiPSC) application, or artificial intelligence technologies used in new drug discovery and development would also have a potent chance or possibility in the clinical treatment or prevention strategies of AD in the future, in addition to the traditional drug candidates in various stages of preclinical or clinical trials of AD.

With the aid of hiPSC technology, the pathological basis of AD has been studied as well as potential treatment options. After using cell culture techniques, hiPSC acquired from AD patients will be developed into neural progenitor cells and neurons [71]. Combining this cell model with gene-editing techniques like CRSIPR-Cas9 offers the ability to quickly assess the properties of possible medications while shedding light on the cellular and molecular illness causes, which can lower the cost and shorten the development cycle for novel therapies. The human iPSC model was also employed to investigate the pathological features of AD in depth and identify potential therapeutic targets for AD medication development in our lab [72]. In addition, human iPSC technology may be employed for individualized treatment like cell transplantation, based on neuro-regeneration therapeutic for AD, to stimulate neuronal and synaptic regeneration in cognition of AD [73], which would provide innovative therapeutic strategy for AD patients.

Additionally, the objective of non-pharmacological treatment is to enhance the daily functioning of AD patients by influencing cognitive function through art therapy or memory training to lessen depressive symptoms and correct sleeping abnormalities [74]. Word games, music therapy, and other activities have been proven in certain studies to be beneficial for training cognitive function. As a result, non-drug therapy can be utilized as a supplement to other methods of treating clinical AD patients. More practical answers for AD prevention techniques will also be provided by outcomes from linked studies on diet, genetics, or environmental variables. Antioxidant mediators, including vitamin E, have been shown to be beneficial at delaying the progression of AD [75, 76].

## CONCLUSION

Therefore, by investigating the unique biomarkers of early diagnosis and advanced stage, it will elucidate the specific pathological process of AD and offer fresh suggestions for clinical treatment techniques, along with significant and valuable scientific research value.

## AUTHOR CONTRIBUTION

Zenghui Teng (Conceptualization; Data curation; Writing – original draft; Writing – review & editing).

## References

[1] Scheltens P , De Strooper B , Kivipelto M , Holstege H , Chételat G , Teunissen CE , Cummings J , van der Flier WM (2021) Alzheimer’s disease. Lancet 397, 1577–1590. 33667416 10.1016/S0140-6736(20)32205-4 PMC8354300

[2] Sierksma A , Escott-Price V , De Strooper B (2020) Translating geneticrisk of Alzheimer’s disease into mechanistic insight and drugtargets. Science 370, 61–66. 33004512 10.1126/science.abb8575

[3] (2022) 2022 Alzheimer’s disease facts and figures. Alzheimers Dement 18, 700–789. 35289055 10.1002/alz.12638

[4] Gauthier S , Rosa-Neto P , Morais JA , Webster C (2021) World Alzheimer Report 2021: Journey through the diagnosis of dementia. Alzheimer’s Disease International, London, UK.

[5] Qiang W , Yau WM , Lu JX , Collinge J , Tycko R (2017) Structuralvariation in amyloid-β fibrils from Alzheimer’s diseaseclinical subtypes. Nature 541, 217–221. 28052060 10.1038/nature20814 PMC5233555

[6] Yamazaki Y , Zhao N , Caulfield TR , Liu CC , Bu G (2019) ApolipoproteinE and Alzheimer disease: Pathobiology and targeting strategies. Nat Rev Neurol 15, 501–518. 31367008 10.1038/s41582-019-0228-7 PMC7055192

[7] Crous-Bou M , Minguillón C , Gramunt N , Molinuevo JL (2017) Alzheimer’s disease prevention: From risk factors to earlyintervention. Alzheimers Res Ther 9, 71. 28899416 10.1186/s13195-017-0297-z PMC5596480

[8] Broom GM , Shaw IC , Rucklidge JJ (2019) The ketogenic diet as apotential treatment and prevention strategy for Alzheimer’s disease. Nutrition 60, 118–121. 30554068 10.1016/j.nut.2018.10.003

[9] Shi Y , Yamada K , Liddelow SA , Smith ST , Zhao L , Luo W , Tsai RM , Spina S , Grinberg LT , Rojas JC , Gallardo G , Wang K , Roh J , Robinson G , Finn MB , Jiang H , Sullivan PM , Baufeld C , Wood MW , Sutphen C , McCue L , Xiong C , Del-Aguila JL , Morris JC , Cruchaga C , Fagan AM , Miller BL , Boxer AL , Seeley WW , Butovsky O , Barres BA , Paul SM , Holtzman DM Alzheimer’s disease Neuroimaging Initiative (2017) ApoE4markedly exacerbates tau-mediated neurodegeneration in a mouse modelof tauopathy. Nature 549, 523–527. 28959956 10.1038/nature24016 PMC5641217

[10] Karran E , De Strooper B (2022) The amyloid hypothesis in Alzheimer disease: New insights from new therapeutics. Nat Rev DrugDiscov 21, 306–318. 10.1038/s41573-022-00391-w 35177833

[11] Otero-Garcia M , Mahajani SU , Wakhloo D , Tang W , Xue YQ , Morabito S , Pan J , Oberhauser J , Madira AE , Shakouri T , Deng Y , Allison T , He Z , Lowry WE , Kawaguchi R , Swarup V , Cobos I (2022) Molecular signaturesunderlying neurofibrillary tangle susceptibility in Alzheimer’s disease. Neuron 110, 2929–2948.e8 35882228 10.1016/j.neuron.2022.06.021 PMC9509477

[12] Busche MA , Hyman BT (2020) Synergy between amyloid-β and tauin Alzheimer’s disease. Nat Neurosci 23, 1183–1193. 32778792 10.1038/s41593-020-0687-6 PMC11831977

[13] Hur JY , Frost GR , Wu X , Crump C , Pan SJ , Wong E , Barros M , Li T , Nie P , Zhai Y , Wang JC , Tcw J , Guo L , McKenzie A , Ming C , Zhou X , Wang M , Sagi Y , Renton AE , Esposito BT , Kim Y , Sadleir KR , Trinh I , Rissman RA , Vassar R , Zhang B , Johnson DS , Masliah E , Greengard P , Goate A , Li YM (2020) The innate immunity protein IFITM3 modulatesγ-secretase in Alzheimer’s disease.. Nature 586, 735–740. 32879487 10.1038/s41586-020-2681-2 PMC7919141

[14] Yang G , Zhou R , Guo X , Yan C , Lei J , Shi Y (2021) Structural basisof γ-secretase inhibition and modulation by small molecule drugs. Cell 184, 521–533e14. 33373587 10.1016/j.cell.2020.11.049

[15] Strömberg K , Eketjäll S , Georgievska B , Tunblad K , Eliason K , Olsson F , Radesäter AC , Klintenberg R , Arvidsson PI , von Berg S , Fälting J , Cowburn RF , Dabrowski M (2015) Combining anamyloid-beta (Aβ) cleaving enzyme inhibitor with aγ-secretase modulator results in an additive reduction ofAβ production. FEBS J 282, 65–73. 25303711 10.1111/febs.13103

[16] Panza F , Lozupone M , Logroscino G , Imbimbo BP (2019) A criticalappraisal of amyloid-β-targeting therapies for Alzheimer disease. Nat Rev Neurol 15, 73–88. 30610216 10.1038/s41582-018-0116-6

[17] Nakamura A , Kaneko N , Villemagne VL , Kato T , Doecke J , Doré V , Fowler C , Li QX , Martins R , Rowe C , Tomita T , Matsuzaki K , Ishii K , Ishii K , Arahata Y , Iwamoto S , Ito K , Tanaka K , Masters CL , Yanagisawa K (2018) High performance plasma amyloid-βbiomarkers for Alzheimer’s disease. Nature 554, 249–254. 29420472 10.1038/nature25456

[18] Yu M , Sporns O , Saykin AJ (2021) The human connectome in Alzheimer disease –relationship to biomarkers and genetics. NatRev Neurol 17, 545–563. 10.1038/s41582-021-00529-1 PMC8403643 34285392

[19] Huang L , McClatchy DB , Maher P , Liang Z , Diedrich JK , Soriano-Castell D , Goldberg J , Shokhirev M , Yates JR 3rd , Schubert D , Currais A (2020) Intracellular amyloid toxicity inducesoxytosis/ferroptosis regulated cell death.. Cell Death Dis 11, 828. 33024077 10.1038/s41419-020-03020-9 PMC7538552

[20] Okazawa H (2021) Intracellular amyloid hypothesis for ultra-earlyphase pathology of Alzheimer’s disease.. Neuropathology 41, 93–98. 33876503 10.1111/neup.12738 PMC8251586

[21] Teng Z , Kartalou GI , Dagar S , Fraering PC , Lessmann V , Gottmann K (2023) A delay in vesicle endocytosis by a C-terminal fragment of N-cadherin enhances Aβ synaptotoxicity. Cell DeathDiscov 9, 444. 10.1038/s41420-023-01739-w PMC10703901 38062019

[22] Rice HC , de Malmazet D , Schreurs A , Frere S , Van Molle I , Volkov AN , Creemers E , Vertkin I , Nys J , Ranaivoson FM , Comoletti D , Savas JN , Remaut H , Balschun D , Wierda KD , Slutsky I , Farrow K , De Strooper B , de Wit J (2019) Secreted amyloid-β precursor proteinfunctions as a GABABR1a ligand to modulate synaptictransmission . . Science 363, eaao4827. 30630900 10.1126/science.aao4827 PMC6366617

[23] Andreyeva A , Nieweg K , Horstmann K , Klapper S , Müller-Schiffmann A , Korth C , Gottmann K (2012) C-terminal fragment of N-cadherinaccelerates synapse destabilization by amyloid-β. Brain 135, 2140–2154. 22637581 10.1093/brain/aws120

[24] Sindi IA , Tannenberg RK , Dodd PR (2014) Role for theneurexin-neuroligin complex in Alzheimer’s disease. NeurobiolAging 35, 746–756. 10.1016/j.neurobiolaging.2013.09.032 24211009

[25] Laakso T , Muggalla P , Kysenius K , Laurén J , Paatero A , Huttunen HJ , Airaksinen MS (2012) LRRTM3 is dispensable for amyloid-βproduction in mice. J Alzheimers Dis 31, 759–764. 22710909 10.3233/JAD-2012-120193

[26] Murray HC , Swanson MEV , Dieriks BV , Turner C , Faull RLM , Curtis MA (2018) Neurochemical characterization of PSA-NCAM^+^ cellsin the human brain and phenotypic quantification in Alzheimer’s disease entorhinal cortex. Neuroscience 372, 289–303. 29429526 10.1016/j.neuroscience.2017.12.019

[27] Jin M , O’Nuallain B , Hong W , Boyd J , Lagomarsino VN , O’Malley TT , Liu W , Vanderburg CR , Frosch MP , Young-Pearse T , Selkoe DJ , Walsh DM (2018) An paradigm to assess potentialanti-Aβ antibodies for Alzheimer’s disease. NatCommun 9, 2676. 10.1038/s41467-018-05068-w PMC6041266 29992960

[28] Yanamandra K , Kfoury N , Jiang H , Mahan TE , Ma S , Maloney SE , Wozniak DF , Diamond MI , Holtzman DM (2013) Anti-tau antibodies that blocktau aggregate seeding markedly decrease pathologyand improve cognition. Neuron 80, 402–414. 24075978 10.1016/j.neuron.2013.07.046 PMC3924573

[29] Choi SH , Bylykbashi E , Chatila ZK , Lee SW , Pulli B , Clemenson GD , Kim E , Rompala A , Oram MK , Asselin C , Aronson J , Zhang C , Miller SJ , Lesinski A , Chen JW , Kim DY , van Praag H , Spiegelman BM , Gage FH , Tanzi RE (2018) Combined adult neurogenesis and BDNF mimic exerciseeffects on cognition in an Alzheimer’s mouse model. Science 361, eaan8821. 30190379 10.1126/science.aan8821 PMC6149542

[30] Cummings J , Lee G , Nahed P , Kambar MEZN , Zhong K , Fonseca J , Taghva K (2022) Alzheimer’s disease drug development pipeline: 2022. Alzheimers Dement (N Y) 8, e12295. 35516416 10.1002/trc2.12295 PMC9066743

[31] Teipel SJ , Cavedo E , Grothe MJ , Lista S , Galluzzi S , Colliot O , Chupin M , Bakardjian H , Dormont D , Dubois B , Hampel H HippocampusStudy Group (2016) Predictors of cognitive decline and treatmentresponse in a clinical trial on suspected prodromal Alzheimer’s disease. Neuropharmacology 108, 128–135. 26876309 10.1016/j.neuropharm.2016.02.005

[32] van der Kant R , Goldstein LSB , Ossenkoppele R (2020) Amyloid-β-independent regulators of tau pathology inAlzheimer disease. Nat Rev Neurosci 21, 21–35. 31780819 10.1038/s41583-019-0240-3

[33] Jagust W (2018) Imaging the evolution and pathophysiology ofAlzheimer disease. Nat Rev Neurosci 19, 687–700. 30266970 10.1038/s41583-018-0067-3 PMC7032048

[34] Rubinski A , Tosun D , Franzmeier N , Neitzel J , Frontzkowski L , Weiner M , Ewers M (2021) Lower cerebral perfusion is associated withtau-PET in the entorhinal cortex across the Alzheimer’s continuum. Neurobiol Aging 102, 111–118. 33765424 10.1016/j.neurobiolaging.2021.02.003 PMC8205941

[35] Mattsson N , Palmqvist S , Stomrud E , Vogel J , Hansson O (2019) Staging β-amyloid pathology with amyloid positron emissiontomography. JAMA Neurol 76, 1319–1329. 31314895 10.1001/jamaneurol.2019.2214 PMC6646987

[36] Ontiveros-Torres MÁ , Labra-Barrios ML , Díaz-Cintra S , Aguilar-Vázquez AR , Moreno-Campuzano S , Flores-Rodríguez P , Luna-Herrera C , Mena R , Perry G , Florán-Garduño B , Luna-Muñoz J , Luna-Arias JP (2016) Fibrillar amyloid-β accumulation triggers an inflammatory mechanism leading to hyperphosphorylation of the carboxyl-terminal end of tau polypeptide in the hippocampal formation of the 3×Tg-AD transgenic mouse. J Alzheimers Dis 52, 243–269. 27031470 10.3233/JAD-150837

[37] Giovinazzo D , Bursac B , Sbodio JI , Nalluru S , Vignane T , Snowman AM , Albacarys LM , Sedlak TW , Torregrossa R , Whiteman M , Filipovic MR , Snyder SH , Paul BD (2021) Hydrogen sulfide is neuroprotective in Alzheimer’s disease by sulfhydrating GSK3β and inhibiting Tau hyperphosphorylation. Proc Natl Acad Sci U S A 118, e2017225118. 33431651 10.1073/pnas.2017225118 PMC7848711

[38] Tell V , Hilbrich I , Holzer M , Totzke F , Schachtele C , Slynko I , Sippl W , Hilgeroth A (2016) Drug development of small-molecule inhibitors of AD-relevant kinases as novel perspective multitargeted approach. Curr Alzheimer Res 13, 1330–1336. 27306698 10.2174/1567205013666160615091821

[39] Bettcher BM , Tansey MG , Dorothée G , Heneka MT (2021) Peripheral and central immune system crosstalk in Alzheimer disease –a research prospectus. Nat Rev Neurol 17, 689–701. 34522039 10.1038/s41582-021-00549-x PMC8439173

[40] Shi Y , Holtzman DM (2018) Interplay between innate immunity and Alzheimer disease: APOE and TREM2 in the spotlight. Nat Rev Immunol 18, 759–772. 30140051 10.1038/s41577-018-0051-1 PMC6425488

[41] Laurent C , Burnouf S , Ferry B , Batalha VL , Coelho JE , Baqi Y , Malik E , Mariciniak E , Parrot S , Van der Jeugd A , Faivre E , Flaten V , Ledent C , D’Hooge R , Sergeant N , Hamdane M , Humez S , Müller CE , Lopes LV , Buée L , Blum D (2016) A2A adenosine receptor deletion is protective in a mouse model of tauopathy. Mol Psychiatry 21, 97–107. 25450226 10.1038/mp.2014.151

[42] Thakur S , Dhapola R , Sarma P , Medhi B , Reddy DH (2023) Neuroinflammation in Alzheimer’s disease: Current progress in molecular signaling and therapeutics. Inflammation 46, 1–17. 35986874 10.1007/s10753-022-01721-1

[43] Liddelow SA , Guttenplan KA , Clarke LE , Bennett FC , Bohlen CJ , Schirmer L , Bennett ML , Münch AE , Chung WS , Peterson TC , Wilton DK , Frouin A , Napier BA , Panicker N , Kumar M , Buckwalter MS , Rowitch DH , Dawson VL , Dawson TM , Stevens B , Barres BA (2017) Neurotoxic reactive astrocytes are induced by activated microglia. Nature 541, 481–487. 28099414 10.1038/nature21029 PMC5404890

[44] Freitag K , Sterczyk N , Wendlinger S , Obermayer B , Schulz J , Farztdinov V , Mülleder M , Ralser M , Houtman J , Fleck L , Braeuning C , Sansevrino R , Hoffmann C , Milovanovic D , Sigrist SJ , Conrad T , Beule D , Heppner FL , Jendrach M (2022) Spermidine reduces neuroinflammation and soluble amyloid beta in an Alzheimer’s disease mouse model. J Neuroinflammation 19, 172. 35780157 10.1186/s12974-022-02534-7 PMC9250727

[45] Yang HS , Zhang C , Carlyle BC , Zhen SY , Trombetta BA , Schultz AP , Pruzin JJ , Fitzpatrick CD , Yau WW , Kirn DR , Rentz DM , Arnold SE , Johnson KA , Sperling RA , Chhatwal JP , Tanzi RE (2022) Plasma IL-12/IFN-γ axis predicts cognitive trajectories in cognitively unimpaired older adults. Alzheimers Dement 18, 645–653. 34160128 10.1002/alz.12399 PMC8695625

[46] McAlpine CS , Park J , Griciuc A , Kim E , Choi SH , Iwamoto Y , Kiss MG , Christie KA , Vinegoni C , Poller WC , Mindur JE , Chan CT , He S , Janssen H , Wong LP , Downey J , Singh S , Anzai A , Kahles F , Jorfi M , Feruglio PF , Sadreyev RI , Weissleder R , Kleinstiver BP , Nahrendorf M , Tanzi RE , Swirski FK (2021) Astrocytic interleukin-3 programs microglia and limits Alzheimer’s disease. Nature 595, 701–706. 34262178 10.1038/s41586-021-03734-6 PMC8934148

[47] Nguyen PT , Dorman LC , Pan S , Vainchtein ID , Han RT , Nakao-Inoue H , Taloma SE , Barron JJ , Molofsky AB , Kheirbek MA , Molofsky AV (2020) Microglial remodeling of the extracellular matrix promotes synapse plasticity. Cell 182, 388–403.e15. 32615087 10.1016/j.cell.2020.05.050 PMC7497728

[48] Teng Z , Gottmann K (2022) Hemisynapse formation between target astrocytes and cortical neuron axons. Front Mol Neurosci 15, 829506. 35386271 10.3389/fnmol.2022.829506 PMC8978633

[49] Saresella M , Marventano I , Piancone F , La Rosa F , Galimberti D , Fenoglio C , Scarpini E , Clerici M (2020) IL-33 and its decoy sST2 in patients with Alzheimer’s disease and mild cognitive impairment. J Neuroinflammation 17, 174. 32505187 10.1186/s12974-020-01806-4 PMC7276088

[50] Chandra A , Dervenoulas G , Politis M ; Alzheimer’s Disease Neuroimaging Initiative (2019) Magnetic resonance imaging in Alzheimer’s disease and mild cognitive impairment. J Neurol 266, 1293–1302. 30120563 10.1007/s00415-018-9016-3 PMC6517561

[51] Leuzy A , Chiotis K , Lemoine L , Gillberg PG , Almkvist O , Rodriguez-Vieitez E , Nordberg A (2019) Tau PET imaging in neurodegenerative tauopathies-still a challenge. Mol Psychiatry 24, 1112–1134. 30635637 10.1038/s41380-018-0342-8 PMC6756230

[52] Colato E , Chiotis K , Ferreira D , Mazrina MS , Lemoine L , Mohanty R , Westman E , Nordberg A , Rodriguez-Vieitez E , Alzheimer’s Disease Neuroimaging Initiative (2021) Assessment of tau pathology as measured by 18F-THK5317 and 18F-Flortaucipir PET and their relation to brain atrophy and cognition in Alzheimer’s disease. J Alzheimers Dis 84, 103–117. 34511502 10.3233/JAD-210614 PMC8609906

[53] Gouilly D , Saint-Aubert L , Ribeiro MJ , Salabert AS , Tauber C , Péran P , Arlicot N , Pariente J , Payoux P (2022) Neuroinflammation PET imaging of the translocator protein (TSPO) in Alzheimer’s disease: An update. Eur J Neurosci 55, 1322–1343. 35083791 10.1111/ejn.15613

[54] Mattsson N , Cullen NC , Andreasson U , Zetterberg H , Blennow K (2019) Association between longitudinal plasma neurofilament light and neurodegeneration in patients with Alzheimer disease. JAMA Neurol 76, 791–799. 31009028 10.1001/jamaneurol.2019.0765 PMC6583067

[55] Preische O , Schultz SA , Apel A , Kuhle J , Kaeser SA , Barro C , Gräber S , Kuder-Buletta E , LaFougere C , Laske C , Vöglein J , Levin J , Masters CL , Martins R , Schofield PR , Rossor MN , Graff-Radford NR , Salloway S , Ghetti B , Ringman JM , Noble JM , Chhatwal J , Goate AM , Benzinger TLS , Morris JC , Bateman RJ , Wang G , Fagan AM , McDade EM , Gordon BA , Jucker M Dominantly Inherited Alzheimer Network (2019) Serum neurofilament dynamics predicts neurodegeneration and clinical progression in presymptomatic Alzheimer’s disease. Nat Med 25, 277–283. 30664784 10.1038/s41591-018-0304-3 PMC6367005

[56] Pereira JB , Janelidze S , Smith R , Mattsson-Carlgren N , Palmqvist S , Teunissen CE , Zetterberg H , Stomrud E , Ashton NJ , Blennow K , Hansson O (2021) Plasma GFAP is an early marker of amyloid-β but not tau pathology in Alzheimer’s disease. Brain 144, 3505–3516. 34259835 10.1093/brain/awab223 PMC8677538

[57] Suárez-Calvet M , Karikari TK , Ashton NJ , Lantero Rodríguez J , Milà-Alomà M , Gispert JD , Salvadó G , Minguillon C , Fauria K , Shekari M , Grau-Rivera O , Arenaza-Urquijo EM , Sala-Vila A , Sánchez-Benavides G , González-de-Echávarri JM , Kollmorgen G , Stoops E , Vanmechelen E , Zetterberg H , Blennow K , Molinuevo JL ALFA Study (2020) Novel tau biomarkers phosphorylated at T181, T217 or T231 rise in the initial stages of the preclinical Alzheimer’s continuum when only subtle changes in Aβ pathology are detected. EMBO Mol Med 12, e12921. 33169916 10.15252/emmm.202012921 PMC7721364

[58] Leuzy A , Janelidze S , Mattsson-Carlgren N , Palmqvist S , Jacobs D , Cicognola C , Stomrud E , Vanmechelen E , Dage JL , Hansson O (2021) Comparing the clinical utility and diagnostic performance of CSF P-Tau181, P-Tau217, and P-Tau231 assays. Neurology 97, e1681–e1694. 34493616 10.1212/WNL.0000000000012727 PMC8605616

[59] Milà-Alomà M , Ashton NJ , Shekari M , Salvadó G , Ortiz-Romero P , Montoliu-Gaya L , Benedet AL , Karikari TK , Lantero-Rodriguez J , Vanmechelen E , Day TA , González-Escalante A , Sánchez-Benavides G , Minguillon C , Fauria K , Molinuevo JL , Dage JL , Zetterberg H , Gispert JD , Suárez-Calvet M , Blennow K (2022) Plasma p-tau231 and p-tau217 as state markers of amyloid-β pathology in preclinical Alzheimer’s disease. Nat Med 28, 1797–1801. 35953717 10.1038/s41591-022-01925-w PMC9499867

[60] Rajpurkar P , Chen E , Banerjee O , Topol EJ (2022) AI in health and medicine. Nat Med 28, 31–38. 35058619 10.1038/s41591-021-01614-0

[61] Ding Y , Sohn JH , Kawczynski MG , Trivedi H , Harnish R , Jenkins NW , Lituiev D , Copeland TP , Aboian MS , Mari Aparici C , Behr SC , Flavell RR , Huang SY , Zalocusky KA , Nardo L , Seo Y , Hawkins RA , Hernandez Pampaloni M , Hadley D , Franc BL (2019) A deep learning model to predict a diagnosis of Alzheimer disease by using ^18^F-FDG PET of the brain. Radiology 290, 456–464. 30398430 10.1148/radiol.2018180958 PMC6358051

[62] Yamada Y , Shinkawa K , Kobayashi M , Badal VD , Glorioso D , Lee EE , Daly R , Nebeker C , Twamley EW , Depp C , Nemoto M , Nemoto K , Kim HC , Arai T , Jeste DV (2022) Automated analysis of drawing process to estimate global cognition in older adults: Preliminary international validation on the US and Japan data sets. JMIR Form Res 6, e37014. 35511253 10.2196/37014 PMC9121219

[63] Meier IB , Buegler M , Harms R , Seixas A , Çöltekin A , Tarnanas I (2021) Using a digital neuro signature to measure longitudinal individual-level change in Alzheimer’s disease: The Altoida large cohort study. NPJ Digit Med 4, 101. 34168269 10.1038/s41746-021-00470-z PMC8225898

[64] Cummings J , Zhou Y , Lee G , Zhong K , Fonseca J , Cheng F (2023) Alzheimer’s disease drug development pipeline: 2023. Alzheimers Dement (N Y) 9, e12385. 37251912 10.1002/trc2.12385 PMC10210334

[65] Vaz M , Silvestre S (2020) Alzheimer’s disease: Recent treatment strategies. . Eur J Pharmacol 887, 173554. 32941929 10.1016/j.ejphar.2020.173554

[66] Sevigny J , Chiao P , Bussière T , Weinreb PH , Williams L , Maier M , Dunstan R , Salloway S , Chen T , Ling Y , O’Gorman J , Qian F , Arastu M , Li M , Chollate S , Brennan MS , Quintero-Monzon O , Scannevin RH , Arnold HM , Engber T , Rhodes K , Ferrero J , Hang Y , Mikulskis A , Grimm J , Hock C , Nitsch RM , Sandrock A (2016) The antibody aducanumab reduces Aβ plaques in Alzheimer’s disease. Nature 537, 50–56. 27582220 10.1038/nature19323

[67] Salloway S , Chalkias S , Barkhof F , Burkett P , Barakos J , Purcell D , Suhy J , Forrestal F , Tian Y , Umans K , Wang G , Singhal P , Budd Haeberlein S , Smirnakis K (2022) Amyloid-related imaging abnormalities in 2 phase 3 studies evaluating aducanumab in patients with early Alzheimer disease. JAMA Neurol 79, 13–21. 34807243 10.1001/jamaneurol.2021.4161 PMC8609465

[68] van Dyck CH , Swanson CJ , Aisen P , Bateman RJ , Chen C , Gee M , Kanekiyo M , Li D , Reyderman L , Cohen S , Froelich L , Katayama S , Sabbagh M , Vellas B , Watson D , Dhadda S , Irizarry M , Kramer LD , Iwatsubo T (2023) Lecanemab in early Alzheimer’s disease. N Engl J Med 388, 9–21. 36449413 10.1056/NEJMoa2212948

[69] Reardon S FDA approves Alzheimer’s drug lecanemab amid safety concerns. Nature (2023) 613, 227–228. 36627422 10.1038/d41586-023-00030-3

[70] Tipton PW (2023) Updates on pharmacological treatment for Alzheimer’s disease. Neurol Neurochir Pol, doi:10.5603/pjnns.96286. 37606550

[71] Penney J , Ralvenius WT , Tsai LH (2020) Modeling Alzheimer’s disease with iPSC-derived brain cells. Mol Psychiatry 25, 148–167. 31391546 10.1038/s41380-019-0468-3 PMC6906186

[72] Nieweg K , Andreyeva A , van Stegen B , Tanriöver G , Gottmann K (2015) Alzheimer’s disease-related amyloid-β induces synaptotoxicity in human iPS cell-derived neurons. Cell Death Dis 6, e1709. 25837485 10.1038/cddis.2015.72 PMC4650541

[73] Duncan T , Valenzuela M (2017) Alzheimer’s disease, dementia, and stem cell therapy.. Stem Cell Res Ther 8, 111. 28494803 10.1186/s13287-017-0567-5 PMC5427593

[74] Guzman-Martinez L , Calfío C , Farias GA , Vilches C , Prieto R , Maccioni RB (2021) New frontiers in the prevention, diagnosis, and treatment of Alzheimer’s disease. J Alzheimers Dis 82, S51–S63. 33523002 10.3233/JAD-201059

[75] Gómez Gallego M , Gómez García J (2017) Music therapy and Alzheimer’s disease: Cognitive, psychological, and behavioural effects. Neurologia 32, 300–308. 26896913 10.1016/j.nrl.2015.12.003

[76] Kivipelto M , Mangialasche F , Ngandu T (2018) Lifestyle interventions to prevent cognitive impairment, dementia and Alzheimer disease. Nat Rev Neurol 14, 653–666. 30291317 10.1038/s41582-018-0070-3

